# Adverse events following single dose treatment of lymphatic filariasis: Observations from a review of the literature

**DOI:** 10.1371/journal.pntd.0006454

**Published:** 2018-05-16

**Authors:** Philip J. Budge, Carly Herbert, Britt J. Andersen, Gary J. Weil

**Affiliations:** 1 Infectious Diseases Division, Department of Medicine, Washington University School of Medicine in St. Louis, St. Louis, Missouri, United States of America; 2 Department of Anthropology, Washington University in St. Louis, St. Louis, Missouri, United States of America; University of Cambridge, UNITED KINGDOM

## Abstract

**Background:**

WHO’s Global Programme to Eliminate Lymphatic Filariasis (LF) uses mass drug administration (MDA) of anthelmintic medications to interrupt LF transmission in endemic areas. Recently, a single dose combination of ivermectin (IVM), diethylcarbamazine (DEC), and albendazole (ALB) was shown to be markedly more effective than the standard two-drug regimens (DEC or IVM, plus ALB) for achieving long-term clearance of microfilaremia.

**Objective and methods:**

To provide context for the results of a large-scale, international safety trial of MDA using triple drug therapy, we searched Ovid Medline for studies published from 1985–2017 that reported adverse events (AEs) following treatment of LF with IVM, DEC, ALB, or any combination of these medications. Studies that reported AE rates by treatment group were included.

**Findings:**

We reviewed 162 published manuscripts, 55 of which met inclusion criteria. Among these, 34 were clinic or hospital-based clinical trials, and 21 were community-based studies. Reported AE rates varied widely. The median AE rate following DEC or IVM treatment was greater than 60% among microfilaremic participants and less than 10% in persons without microfilaremia. The most common AEs reported were fever, headache, myalgia or arthralgia, fatigue, and malaise.

**Interpretation:**

Mild to moderate systemic AEs related to death of microfilariae are common following LF treatment. Post-treatment AEs are transient and rarely severe or serious. Comparison of AE rates from different community studies is difficult due to inconsistent AE reporting, varied infection rates, and varied intensity of follow-up. A more uniform approach for assessing and reporting AEs in LF community treatment studies would be helpful.

## Author summary

WHO’s Global Programme to Eliminate Lymphatic Filariais (LF) supports annual mass drug administration to over 400 million people in LF-endemic areas each year. Two drug combinations (either DEC or ivermectin, given with albendazole) have been recommended in most endemic areas. With the exception of well-described serious adverse events (AEs) occurring in patients with high level loiasis, severe AEs due to these medications are extremely rare. Mild to moderate AEs, however, are common, particularly in patients with active filarial infection. In this manuscript we synthesize published data on AEs following single-dose treatment of LF with ivermectin, DEC, or albendazole. This provides a background against which to compare the safety of triple drug therapy (ivermectin, DEC, and albendazole) recently endorsed by WHO, and provides a useful context for evaluating safety of new treatments for LF. The compiled data illustrate that transient, mild to moderate AEs following single-dose LF treatment are common in microfilaremic patients and are much less common in amicrofilaremic patients. They also show that passive surveillance for post-treatment AEs underestimates AE incidence and suggest that adherence to common reporting standards would improve the usefulness of AE reporting in filariasis studies.

## Introduction

Infection with the filarial nematode parasites *Wuchereria bancrofti*, *Brugia malayi*, or *Brugia timori* is known as lymphatic filariasis (LF). These infections cause severe, disabling conditions including lymphedema, elephantiasis, and hydroceles in tens of millions of people in tropical and subtropical countries. Annual mass drug administration (MDA) coordinated by WHO’s Global Programme to Eliminate LF (GPELF) has significantly reduced LF transmission in many of the 78 initially endemic nations [1–3]. Yet LF remains far too common, with tens of millions infected and 850 million people at risk of acquiring the infection in 53 countries [3]. With approximately 500 million people receiving MDA for LF each year, understanding, anticipating, and preparing the targeted population for MDA-related adverse events (AEs) is important for program success.

Medications used for MDA include diethylcarbamazine (DEC), ivermectin (IVM) and albendazole (ALB). The combination of IVM plus ALB is used in areas of Africa where onchocerciasis (river blindness) is co-endemic with LF. Twice yearly ALB alone is recommended for LF-endemic areas of Africa that are co-endemic for loiasis, and DEC plus ALB is used in the rest of the world. Serious (life-threatening) AEs due to MDA are exceedingly rare [4–7]. However, when they do occur they can profoundly impact the treated community and jeopardize program success [8]. When communities are well-informed about the type and severity of AEs to be expected, they may be less likely to avoid MDA out of fear of AEs. Furthermore, the knowledgeability of community health workers (drug distributors) can be a major determinant of MDA adherence [8]. A clear understanding of the nature of expected AEs should empower program managers and community health workers to prepare their communities to anticipate and accept transient AEs, which may in turn improve compliance with MDA and facilitate LF elimination efforts.

A promising new combination therapy for LF that combines a single dose of IVM, DEC, and ALB (IDA) appears to be highly effective [9], and its safety is has been evaluated in large community-based studies in several locations (ClinicalTrials.gov Identifier: NCT02899936) [10]. This manuscript’s purpose is to provide context for understanding the safety of the new IDA treatment by reviewing published data on the rates and nature of AEs following single-dose treatment for LF with any of the IDA medications. As previously noted by many others, AE reporting in LF treatment trials is highly variable and potentially affected by multiple factors including blood microfilaria (Mf) counts, treatment regimens, filarial species, population demographics, and importantly, the thoroughness of post-treatment surveillance. We therefore sought to review AE data from published LF treatment studies to further understand the effect of these parameters on AE rates and severity. Our objective was to evaluate reports of AEs following single dose LF treatment of children and adults with IVM, DEC, or ALB (either as monotherapy or in multidrug combination regimens), published since 1985. In this report we first present a broad summary of the literature reviewed and then a quantitative synthesis of published AE rates from studies meeting our specified inclusion criteria.

## Methods

The primary outcome of interest for the quantitative synthesis was the proportion of participants experiencing at least one AE (aggregate AE rate). Rates of individual AEs were a secondary outcome. We did not use a pre-specified AE definition, but rather accepted all AEs reported by the authors of the individual studies. In this manuscript we classify AEs as mild if they do not interfere with normal daily routine (work or school), as moderate if they interfere with daily routines (work or school) but not with activities of daily living, and as severe if they interfere with activities of daily living or cause temporary incapacitation. These designations correspond to Common Terminology Criteria for AEs grades 1 (mild), 2 (moderate), and 3 (severe). Serious AEs are those that are life-threatening or result in hospitalization or permanent injury (grade 4) or are fatal (grade 5) [11].

### Literature search

We reviewed AE data from studies of LF treatment with single-dose regimens that were published between 1985 and 2017. We searched Ovid Medline and Embase for any articles with Medical Subject Headings (MeSH) terms “Elephantiasis, Filarial” and “Drug Therapy” plus any of the following terms: “Adverse Events”, “Poisoning”, or “Toxicity”. We limited our search to English or French language manuscripts dealing with human infections. The most recent search was conducted on 21 Aug, 2017. Two authors (PB and CH) reviewed each publication and gathered additional pertinent publications from articles referenced therein. Publications with sufficient AE data were selected for a quantitative analysis of AEs as described below. We did not pre-specify nor register a review protocol. We did not attempt to contact authors to identify additional studies.

### Selection of studies for quantitative analysis

Studies published after 1985 that reported AE rates following single-dose LF treatment with IVM, DEC, or ALB (alone or in combination) were included. Studies dealing with multi-day courses (generally of DEC) were reviewed, but excluded from the quantitative analysis, as were studies that either provided inadequate information on AEs by treatment arm or did not conduct AE surveillance within one week following treatment. Complete inclusion and exclusion criteria are shown in **Table 1**. We followed the PRISMA Statement for Reporting Systematic Reviews and Meta-Analysis [12]; the completed PRISMA checklist is available as a supplemental file (**S1 Table**).

**Table 1:** Inclusion and exclusion criteria for quantitative analysis.

Inclusion Criteria	Exclusion Criteria
• Treatment with DEC, IVM, or ALB • Report AE rates by study arm • AE data collected within first week after treatment • Published between 1985 and 2017 • Study arms with ≥ 10 participants	• Studies of multi-dose treatments* • Adverse event assessments conducted long after treatment • Co-infection with *Onchocerca volvulus* or *Loa loa* • Treatment with other antifilarial medications (i.e. doxycycline) • Co-administration of other medications (such as azithromycin or praziquantel)

*We included studies that used a preliminary clearing dose [13–16] or when a “single dose” treatment was split over two days [17].

### Quantitative synthesis

From studies meeting inclusion criteria we extracted data including: study location (country), age range and gender of participants, intensity of surveillance, treatment regimen, Mf prevalence, geometric mean Mf counts, presence of co-infections, overall rate of AEs, and rates for any specific AEs reported. For studies that reported AEs following multiple MDA treatment rounds, we included only the AE rates that occurred after the first treatment. For studies in which one but not all treatment arms met inclusion criteria (for example, when single dose IVM or DEC was compared to 12 days of DEC), we included data only from the arm(s) meeting inclusion criteria. The number of participants reported in our analysis is the number for whom AE surveillance was conducted, which was sometimes lower than the total number treated. For example, one study conducted active post-MDA surveillance within a subset of 483 persons among 8 million people treated [18]; in our analysis, the N for this study was 483.

All extracted data were analyzed using Stata version 12.1 (College Station, TX). Because the data were not normally distributed, we report means and interquartile ranges (IQR) and use boxplots for graphical representation. Since AE reporting was insufficiently uniform among included studies, we did not attempt a formal meta-analysis of AE rates, nor did we attempt statistical analyses. Rather, we sought to present a graphical synthesis of data from these disparate studies to illustrate the range of data and an estimate of central tendency (median and interquartile range). To assess for reporting bias in individual studies, we stratified surveillance for AEs in each study as active (individual participants were contacted and asked about AEs) or passive (individuals with AEs had to seek out the study team to report). The quality of active surveillance was further categorized as “high” (at least daily contact during the first 72 hours), “moderate” (at least one contact within first 72 hours), or “low” (participants contacted after 72 hours). Although we hoped to analyze the effect of each extracted variable on reported AE rates, we found that the quality of data reported for most parameters was insufficient. We therefore limited our analysis to an ad hoc comparison of treatment regimens, Mf status, and intensity of AE surveillance.

## Results

### Literature review

Many informative articles that reported AEs following treatment for LF could not be included in our quantitative analysis either because they reported composite AE scores rather than rates, or because they did not report AE rates separately by treatment group. We have attempted to review some of the observations from both included and excluded publications in the following paragraphs.

#### LF MDA medications

Diethylcarbamazine (DEC). DEC is a piperazine derivative with microfilaricidal and partial macrofilaricidal activity [19], and the amount traditionally given for LF treatment was 72 mg/kg divided in 12 daily doses. Although some early studies suggested that weekly or monthly treatments might be equally or more effective than the 12 day treatment course [20, 21], it was not until single dose IVM was shown to drastically reduce Mf counts that single dose DEC was tested and found to be effective [16, 22–24]. A 6mg/kg DEC dose appears to have the best balance between efficacy and AEs [22]. Mf counts drop markedly in the first few days following single-dose DEC with continued slow decline for 6 or 7 months. This decline is generally followed by partial rebound of microfilaremia [16, 24–30]; the level to which Mf counts rebound may be lower with higher cumulative DEC doses [19, 31–36]. Serious AEs including loss of vision and fatal encephalopathy can occur when DEC is given to persons with active onchocerciasis or loiasis [37]. DEC is therefore not used in areas of Africa where LF is co-endemic with *O*. *volvulus* or *L*. *loa*. Direct pharmacologic AEs in persons with no filarial infections are generally limited to transient gastrointestinal upset (nausea, vomiting, diarrhea), dizziness, or lightheadedness that occur within a few hours of ingestion [19, 38].

Ivermectin (IVM). IVM is a semisynthetic member of the avermectin class of antihelminthics initially isolated from fermentation products of *Streptomyces avermitilis*. IVM treatment for LF was first reported in the late 1980s [39–41] and 15 early safety and efficacy studies of IVM for bancroftian filariasis were reviewed in a 1997 meta analysis [42]. IVM dosing for LF MDA is based on height and roughly corresponds to 150–200 μg/kg. Single dose IVM induces a rapid clearance of circulating Mf within the first five days; rebound of microfilaremia following IVM begins as early as 30 days post-treatment with continued increases out to 6 months [16, 25, 40, 41, 43–46]. This rapid Mf clearance occurs with doses as low as 20 μg/kg [15, 39, 46], but higher doses (200 to 400 μg/kg) may have a more prolonged effect [47]. Unlike DEC, single dose IVM has little or no effect on filarial antigen levels [48–50], suggesting a lack of macrofilaricidal effect. IVM is teratogenic at repeated high doses in laboratory animals and is not given to children weighing less than 15 kg or to pregnant women during MDA for LF [2, 51]. As with DEC, IVM can precipitate severe encephalopathy and death when given to persons heavily infected with *L*. *loa* [52, 53], and is not used as MDA for LF in areas with high intensity *L*. *loa* infections.

Albendazole (ALB). ALB belongs to the benzimidazole group of anthelminthic agents and is thought to inhibit tubulin polymerization leading to immobilization and death of susceptible helminths. Two meta-analyses of ALB combination therapy for LF published in 2005 disagreed regarding ALB’s value for treating LF [6, 7], and its inclusion in LF MDA regimens is partially due to its activity against soil-transmitted helminths (STH) [6, 7, 54]. Single-dose ALB alone induces very slow declines in Mf counts and a minor decrease in filarial antigen levels, suggesting a partial macrofilaricidal effect [54–56], which may be enhanced by twice yearly treatment [57, 58]. Although high dose ALB (400 mg twice daily) for 3 weeks induced scrotal reactions in 11 of 15 men in one study (compared to zero of 13 DEC-treated men) [59], there is no evidence that adding single-dose ALB to IVM or DEC for LF MDA increases AEs [5]. In areas where LF is co-endemic with loiasis, semiannual ALB alone (together with integrated vector management) is recommended for LF MDA, because ALB can be safely given to persons with loiasis [60, 61].

#### AEs following treatment of LF

Transient mild to moderate adverse reactions such as fever, headache, dizziness, malaise, myalgia, fatigue, and gastrointestinal upset are common after treatment of LF and are primarily related to dying Mf [5, 6, 62]. Less commonly reported AEs include cough and dyspnea [15, 17, 23, 40] (sometimes associated with blood-tinged sputum and transient pulmonary infiltrates [63] or bronchoconstriction [40, 64]), urticaria or other rash [45, 63, 65], transient proteinuria or hematuria [9, 44, 63, 66], elevated alkaline phosphatase [63] or transaminase levels [46, 59, 65], palpebral edema (with DEC) [17], increased eosinophilia 7–14 days post-treatment [17, 40, 59, 67], and postural hypotension [16, 23, 40, 45, 68]. Systemic AEs generally occur with 24–48 hours after the first dose of microfilaricidal medications (IVM or DEC) [23, 24, 29, 30, 40, 43–45, 54, 56, 64, 67–75], including when low “clearing doses” of drugs are given before full therapeutic doses [14, 15, 24]. Most systemic AEs resolve within 48 hours of onset, although they sometimes last longer [15, 16, 24, 29, 30, 40, 44, 45, 54, 56, 64, 67, 69, 73, 75].

Localized AEs following LF treatment are much less common than systemic AEs. They include the development of subcutaneous or scrotal nodules, spermatic cord swelling, new onset or increased hydrocele, dilated and inflamed lymphatic vessels (the “string sign”) [68], arthritis, lymphadenitis (sometimes with suppuration), and new onset lymphedema [76]. Local reactions may occur within 24–48 hours after treatment, but often the onset is later (1 week or more after treatment) [15, 17, 44, 45, 55, 59, 66, 72, 77]. They are generally self-limited, resolving over the course of one to several weeks. They are thought to be caused by the death of adult filarial worms in lymphatic vessels [14, 15, 17, 19, 44, 63, 78, 79]; prior to the development of adult worm antigen assays and ultrasound visualization of adult worms [79], rates of scrotal reactions were frequently used as a surrogate measure of macrofilaricidal activity [15, 59, 77]. Biopsies of scrotal nodules confirm the presence of dead or dying adult worms and excised nodules sometimes contain both dying and healthy adult worms [79–81].

#### Factors potentially affecting reported AE rates

Infection intensity. Because AEs are related to the death of circulating Mf, the rates and severity of AEs following LF treatment increase with increasing Mf loads and increased microfilaricidal efficacy [14–16, 22, 30, 34, 38, 39, 43, 47, 54, 65, 78, 82–84]. Because of this, AEs are highest following the first dose of microfilaricidal drugs, and they tend to be less frequent and milder in later treatment rounds [47, 67, 74, 77, 85–89]. One notable exception was an initial dose-finding study of IVM against brugian filariasis that did not observe a correlation between AEs and pre-treatment Mf counts [67].

Gender and age. Most studies that reported gender-specific rates of directly-observed AEs did not report significantly different rates between men and women [15, 22, 30, 56, 90, 91]. However, two community MDA studies with passive AE surveillance reported higher rates in women. This may reflect gender-specific differences in reporting, rather than physiologic differences [76, 92]. In one large MDA study in Haiti, moderate AEs (those interfering with school or work) were more frequently reported by men, and these were most commonly due to scrotal pain [72]. Reported AE rates tend to be lower in children [48, 49, 54, 64, 84].

Species differences. Differences in susceptibility to treatment between brugian and bancroftian filariasis may affect AE rates among those who are Mf positive. For bancroftian filariasis, IVM appears to clear Mf more rapidly and more completely than DEC [14, 16, 17, 25, 30, 45, 93, 94], but Mf counts also rebound sooner following IVM treatment. DEC induces a slower, more sustained decline, with gradual Mf reductions out to about 6 months [25, 31, 64, 95]. Over a longer period, reductions in Mf counts are equivalent [14, 25, 71, 96, 97] or slightly better with DEC [16, 17, 24, 45]. For bancroftian filariasis, acute reactions tend to be higher with IVM and later (localized) reactions higher with DEC [17, 45].

The decline in Mf counts following IVM treatment may be somewhat slower in persons with brugian filariasis [65, 67] and AEs following IVM treatment of brugian filariasis may be less severe and occur slightly later post-treatment (day 2–3) [65]. *Brugia* Mf may be more sensitive to DEC-mediated killing [19, 24, 68, 82], and *Brugia* Mf carriers may have more AEs when treated with DEC than *W*. *bancrofti* Mf carriers [24, 73, 82]. Treatment of brugian filariasis does not result in scrotal reactions, a finding that reflects *Brugia*’s lack of tropism for scrotal lymphatic vessels and the lack of hydroceles in brugian filariasis [24].

Medication dose. The initial evaluation of IVM for bancroftian filariasis suggested that 25 μg/kg worked as well as higher doses, but with fewer AEs [40]. The initial study of IVM for brugian filariasis also had a trend towards lower AEs with 25 μg/kg, but this was not statistically significant [67]. In general, however, the level of microfilaremia is much more predictive of AEs than the dose of antifilarial medication [39, 84]. For example, in several studies where low “clearing doses” of medications were given prior to higher “treatment” doses, AE rates were higher with the clearing dose than with the treatment dose for both IVM [13, 15, 98] and DEC [98].

Passive vs. active surveillance. Intensity of AE surveillance following MDA can have a large effect on reported AE rates. One study directly compared AE rates reported by active surveillance (daily visits) with AE reports from subsequent coverage surveys and found 3–4 fold higher AE rates identified by active surveillance [18]. In passive surveillance studies, the rate of reported symptoms may have more to do with general health-seeking behavior than with Mf-related AEs [99].

Co-infections. Co-infections with other filarial species can have dramatic effects on AEs experienced following treatment for LF. These include the well-recognized risk of ocular damage with visual loss after treatment of onchocerciasis with DEC, and encephalopathy and death in persons with heavy loiasis infections [52, 53]. GI symptoms following MDA for LF may be partially related to the effects of treatment on intestinal helminths; extremely low rates of GI side effects were observed in one study that excluded those with a positive stool ova and parasite screen from treatment [56].

#### AE pathogenesis

*Wolbachia*. The molecular mechanisms underlying AEs following LF treatment are incompletely understood, but one leading hypothesis is that they are related to release of bacterial endotoxin-like lipoproteins from endosymbiotic *Wolbachia* released from dying Mf. Several lines of evidence support this hypothesis. First, *B*. *malayi-*infected adults with moderate to severe AEs were more likely to have *Wolbachia* DNA in post-treatment serum samples compared to those with mild or no AEs [100]. Second, a lipopolysaccharide (LPS)-like activity in *B*. *malayi* extract is a potent inducer of TNF-α, IL-1ß and nitric oxide (NO) in murine macrophages, while extracts from the rodent filarial parasite *A*. *viteae* (which do not harbor *Wolbachia*) do not induce these responses [101, 102]. It was surprising, therefore, when the *B*. *malayi*-associated *Wolbachia* genome published in 2004 did not contain genes required for the biosynthesis of lipid A, an essential component of LPS [103]. This suggested that *Wolbachia* does not contain LPS in its cell wall [104]. However, a peptidoglycan-associated lipoprotein (PAL) in *Wolbachia* may effectively mimic the activity of LPS. PAL can signal through TLR2/TLR6, induces pro-inflammatory responses *in vitro* (in murine and human cells) and *in vivo* (mice) [105], and is abundant in *B*. *malayi*, where it localizes to the *Wolbachia* membrane [106]. Finally, depletion of *Wolbachia* with a three or six week treatment course of doxycycline prior to anti-filarial treatment with ALB plus either DEC or IVM decreased the risk of acute AEs [107, 108]; whether this was due to depletion of *Wolbachia* or to doxycycline-related reductions in Mf counts is unclear. Concurrent treatment with doxycycline and DEC reduced inflammatory cytokines and AE severity among microfilaremic patients in another study [109], but an imbalance in the baseline Mf counts in the latter study makes that finding difficult to interpret. Another study did not demonstrate a significant reduction in AEs in persons with LF after pre-clearance of *Wolbachia* with doxycycline [110].

Circulating immune complexes. Circulating immune complexes may also contribute to the development of AEs following LF treatment. These are heterogeneous aggregates of antigens, antibodies and components of the complement cascade [111] that activate pro-inflammatory pathways when they circulate or accumulate in tissue. One publication reported that filarial excretory-secretory antigens and immune complex titers increased after DEC treatment of LF patients [112]. A later study showed that these changes were temporally correlated with the onset of AEs and that their magnitude was significantly greater in individuals who developed AEs after treatment [113].

### Quantitative synthesis of published AE rates

**Fig 1:**
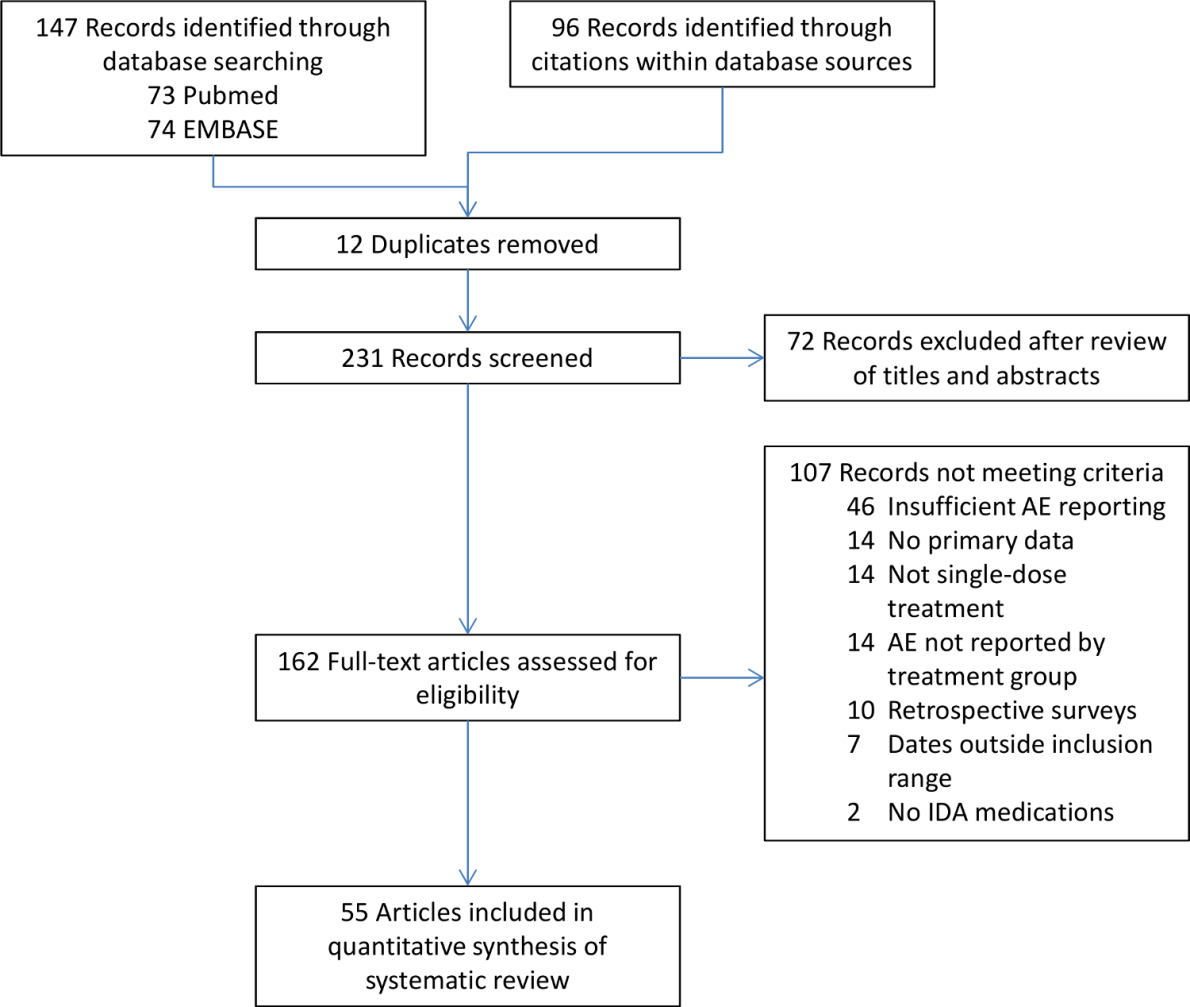
PRISMA flow diagram for inclusion of articles in the quantitative synthesis.

To summarize published rates of AEs following single-dose treatment of LF, and to explore how these might differ by treatment medication and AE surveillance, we compiled data from articles with adequate AE reporting into a combined analysis. Among 162 full text articles reviewed, 55 contained AE data that met criteria for inclusion (**Fig 1**). There was considerable heterogeneity in the way that AEs were reported in these studies; 34 reported both the aggregate incidence of AEs (i.e. the number of persons experiencing any AE) and the percentage of persons experiencing specific AEs. Seventeen studies reported an aggregate incidence but not specific events, and four reported specific events but not aggregate incidence. Methods of AE ascertainment varied widely between studies, from intensive in-hospital monitoring to passive reporting in community-based trials. For the purposes of our analyses we grouped the studies into two main types: (1) clinical trials with active AE surveillance and (2) community studies with either active or passive surveillance. The former group comprises studies in which 100% of participants were Mf positive, while community studies had varied Mf rates (**Table 2**).

**Table 2:** Articles included in the quantitative analysis by article type and study location.

Study location	N Studies	N participants	References
Hospital or Clinic Based Studies with High Quality, Active Surveillance			
Brazil	3	194	[17, 44, 64]
China	1	60	[71]
Egypt	1	71	[45]
French Polynesia	7	314	[69, 85, 90, 93, 94, 114, 115]
Haiti	4	340	[13, 15, 54, 84]
India	11	591	[16, 30, 40, 43, 56, 67, 68, 75, 87, 116, 117]
Indonesia	1	15	[23]
Malaysia	1	40	[65]
Papua New Guinea	1	24	[9]
Senegal	1	16	[41]
Sri Lanka	2	77	[14, 46]
Tanzania	1	25	[118]
**Subtotal**	**34**	**1,767**	
Community Based Studies with Active Surveillance			
Brazil	2	818	[76, 119]
Egypt	1	28	[77]
French Polynesia	2	4,421	[39, 47]
Ghana	2	1,299	[108, 120]
India	5	10,596	[18, 86, 110, 121, 122]
Papua New Guinea	1	966	[123]
Samoa	1	458	[22]
Sri Lanka	1	31	[29]
Tanzania	1	57	[124]
**Subtotal**	**16**	**18,674**	
Community Based Studies with Passive Surveillance			
Ghana	1	15,020	[125]
Haiti	2	74,968	[72, 99]
Kenya	1	170	[126]
Mali	1	42	[127]
**Subtotal**	**5**	**90,200**	
**Total**	**55**	**110,641**	

#### AE rates by Mf status and surveillance intensity

**Fig 2:**
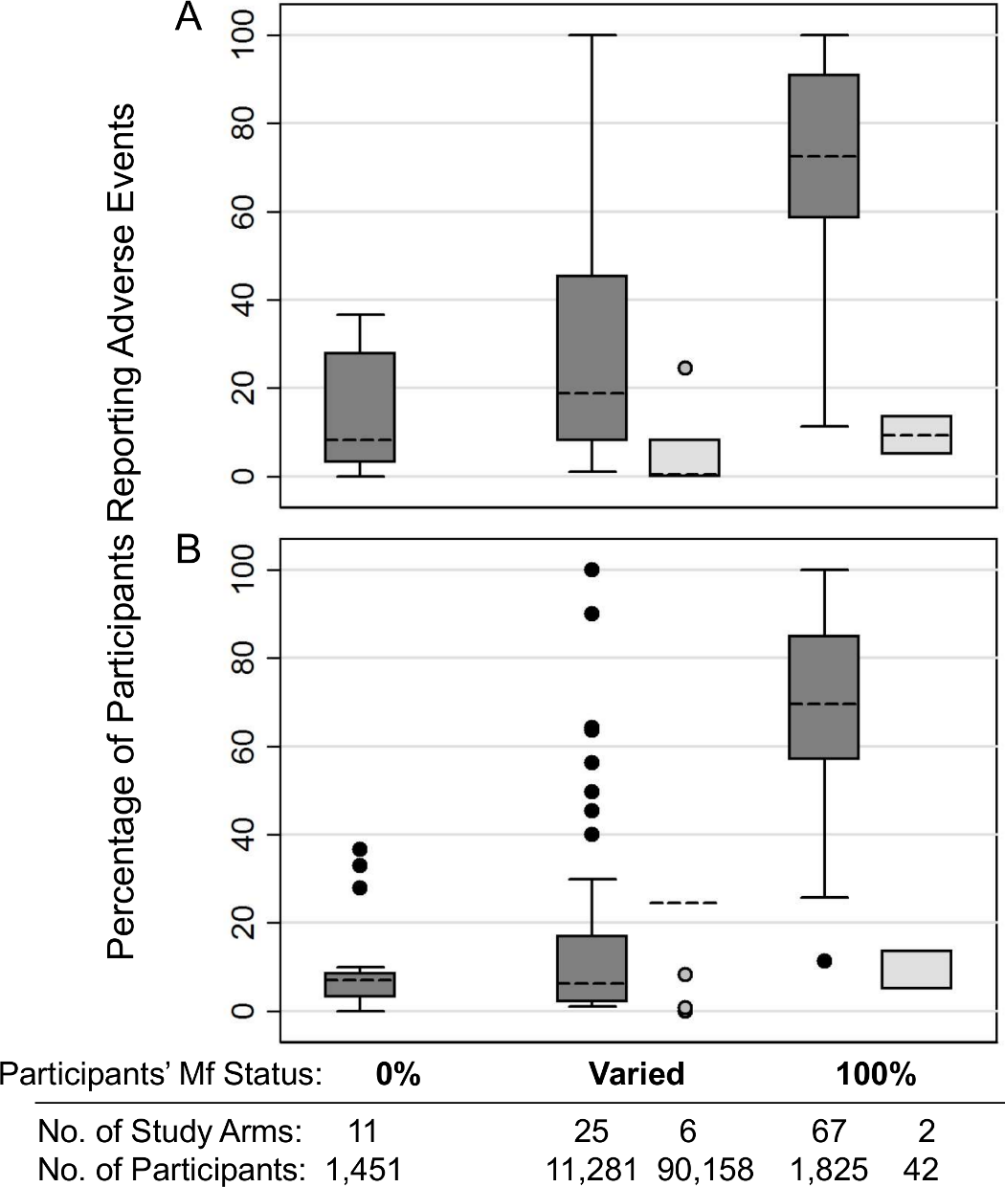
Box plot of reported percentage of participants experiencing at least one AE among individual treatment groups (study arms), comparing active (dark boxes) and passive (light boxes) surveillance methods, excluding placebos. Groups where no participants (0%) or all participants (100%) were Mf positive were from hospital or clinic-based clinical trials; those with “varied” Mf status were from community studies. A) Results when each study group was considered to have equal weight. B) Results when results were adjusted to consider the number of participants in each study group. Boxes indicate the interquartile range (IQR) and dashed line the median. Whiskers indicate the upper and lower adjacent values: upper = the greatest value less than (75th percentile + (1.5 x IQR)); lower = the lowest value greater than (25th percentile–(1.5 x IQR)).

As shown in **Fig 2**, reported AE rates were markedly higher in study arms where 100% of participants were Mf positive. The median aggregate AE rate (proportion of participants experiencing at least one AE) among study arms where all participants were microfilaremic was 70% when each study was given equal weight and 67% when results were adjusted to consider the number of participants per study arm. Reported AE rates in non-placebo groups with no microfilaremia ranged from zero to 37% with a median of 8.3% (7.1% when adjusted for participants per study arm). As expected, studies with passive surveillance reported much lower AE rates (ranging from 0 to 24% regardless of Mf prevalence); this included two study arms from one study in which all participants were microfilaremic [127]. A complete list of the studies included with the extracted data and notes is available as a supplement (**S2 Table**).

#### AE rates by treatment

**Fig 3:**
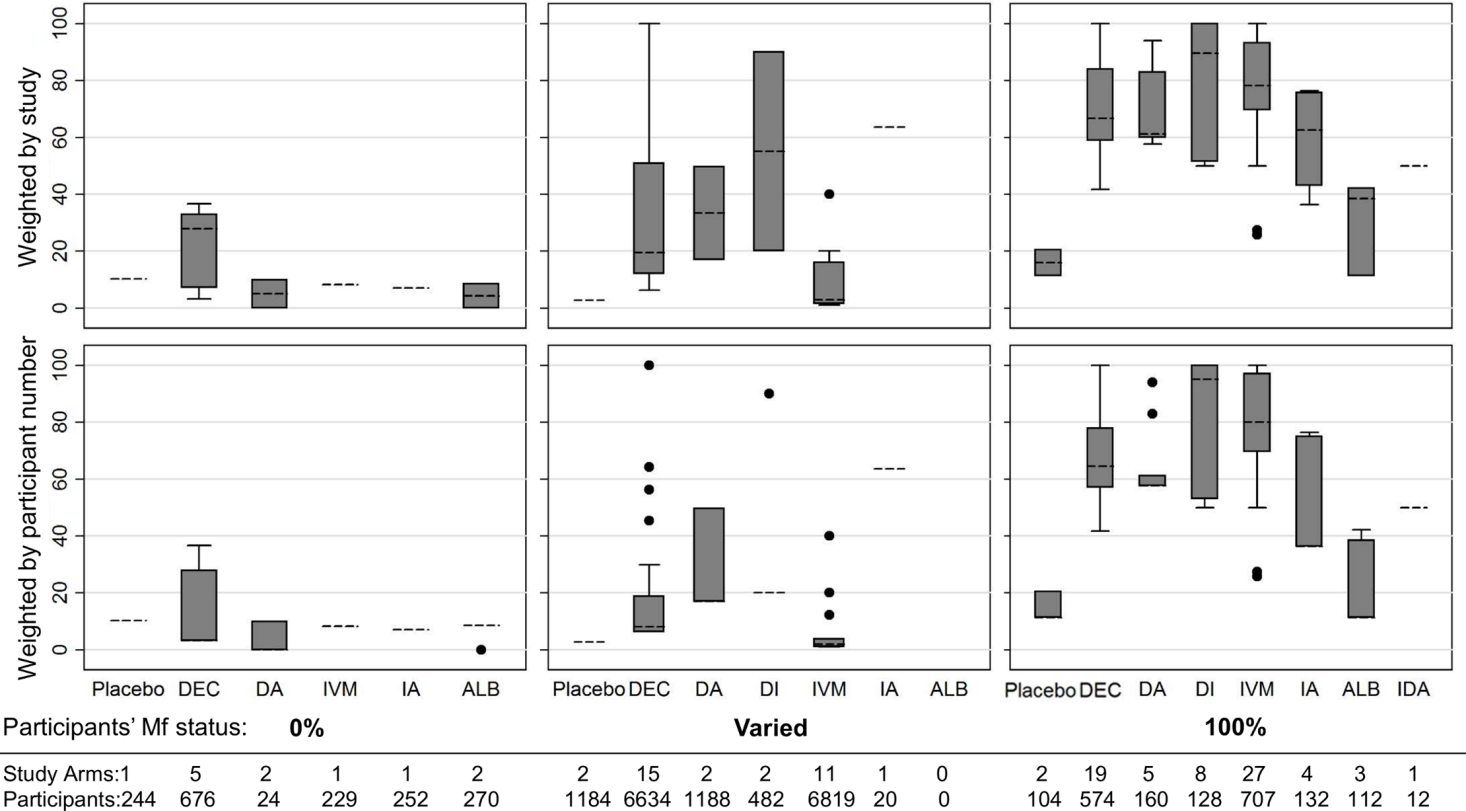
Percentage of participants experiencing at least one AE in studies with active surveillance, according to the microfilaremia (Mf) status of participants. Groups where no participants (0%) or all participants (100%) were Mf positive were from hospital or clinic-based clinical trials; those with “varied” Mf status were from community studies. The top panels show equal weighting for each study, regardless of participant number; results in the bottom panels are weighted by participant number. Boxes indicate the interquartile range (IQR) and dashed line the median. Whiskers indicate the upper and lower adjacent values: upper = the greatest value less than (75th percentile + (1.5 x IQR)); lower = the lowest value greater than (25th percentile–(1.5 x IQR). Abbreviations: ALB = Albendazole, DA = DEC + Albendazole, DI = DEC + Ivermectin, IA = Ivermectin + Albendazole, IDA = Ivermectin + DEC + Albendazole, IVM = Ivermectin.

Aggregated AE rates by treatment regimen from studies with active surveillance are shown in **Fig 3**. AE rates in amicrofilaremic groups were less than or equal to 10% in all groups except among persons treated with DEC only. Among the five included amicrofilaremic treatment groups receiving DEC, three were from a single study that compared different DEC doses (4, 6, or 8 mg/kg) and reported AE rates >25% with all three DEC doses [22]. It is difficult to explain the unusually high AE rates reported from that study.

Aggregate AE rates after treatment of *microfilaremic* persons with regimens that included DEC or IVM ranged between 40 and 100%, with two exceptions: Dunyo reported AE rates of 25.8% and 36.3% among those treated with IVM alone or IVM plus ALB, respectively [120], and Mak reported a rate of 27.5% among those receiving IVM alone [65]. Among the three included studies reporting aggregate AE rates after ALB only treatment, AEs were reported in 11% of 80 participants (tactile fever, myalgia, headache, weakness, abdominal pain, itching) [120], 38% of 12 participants (itching, palpitation, or fever) [118], and 42% of 19 participants (predominantly fever and myalgia) [56].

#### Rate of specific AEs

**Fig 4:**
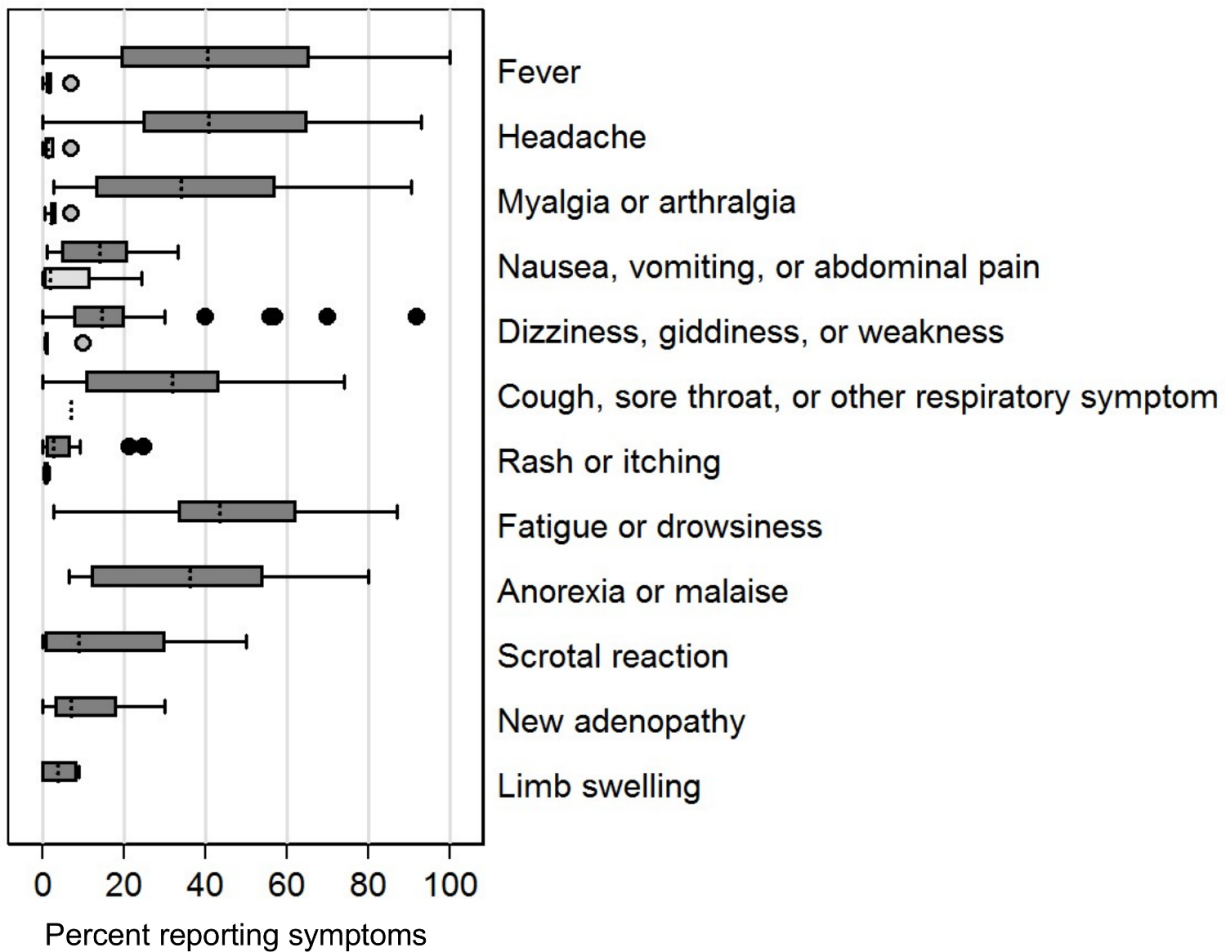
Reported percentages of specific AEs in treatment arms with 100% microfilaremia rates (shaded bars) or no microfilaremia (open bars). Only studies with active AE reporting are included and each study arm is given equal weight (not weighted by participant number). Boxes indicate the interquartile range (IQR) and dashed line the median. Whiskers indicate the upper and lower adjacent values: upper = the greatest value less than (75th percentile + (1.5 x IQR)); lower = the lowest value greater than (25th percentile–(1.5 x IQR)).

**Fig 5:**
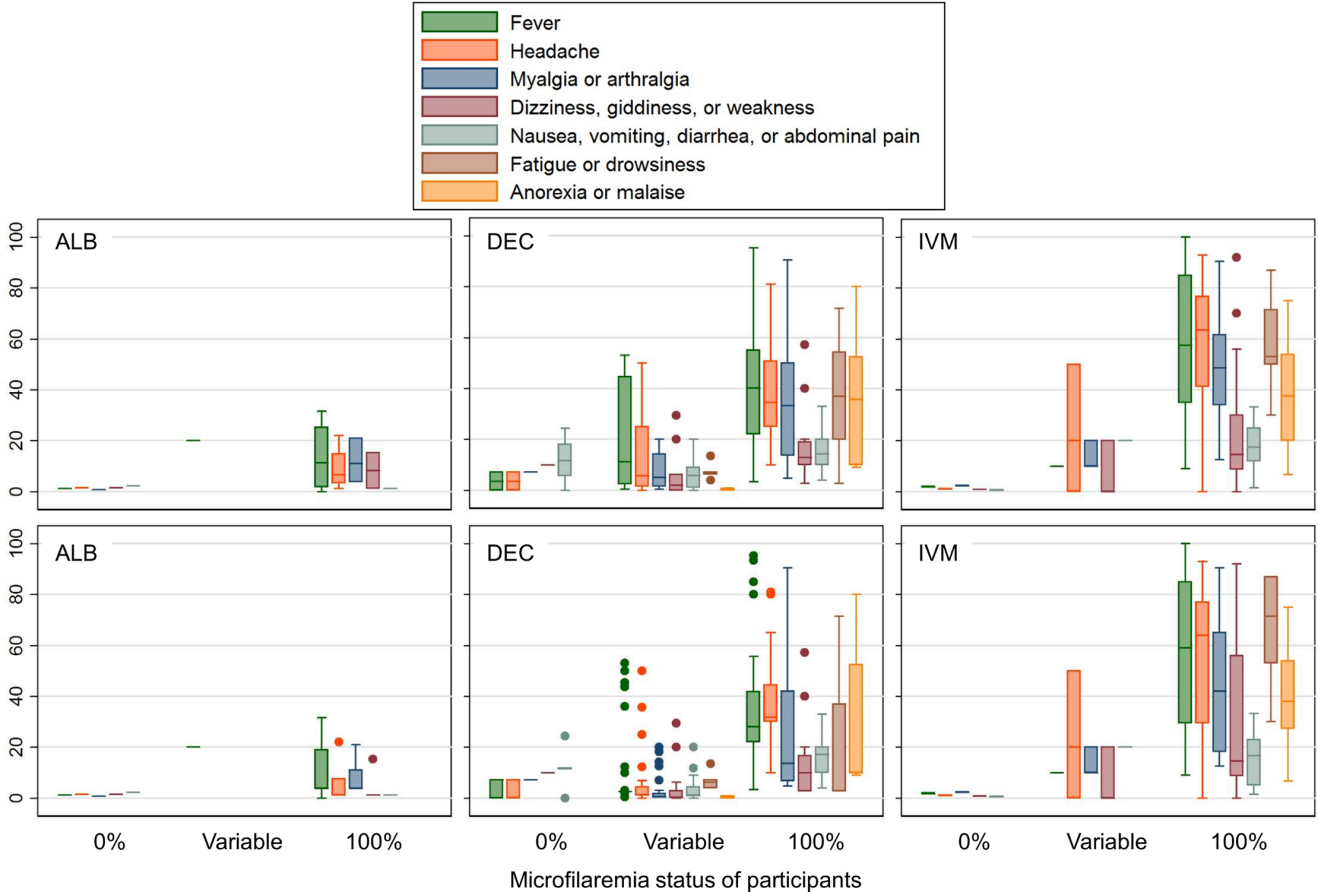
Percentage of participants experiencing common systemic AEs by treatment group, according to the microfilaremia status of participants. DEC and IVM data include all study arms where the indicated drug was given alone or in combination (with or without ALB). For clarity, the ALB panel shows only study arms in which ALB alone was given. The upper panels assign each study are equal weight; the lower panels are weighted according to number of participants per study arm. Boxes indicate the interquartile range (IQR) and horizontal line the median. Whiskers indicate the upper and lower adjacent values: upper = the greatest value less than (75th percentile + (1.5 x IQR)); lower = the lowest value greater than (25th percentile–(1.5 x IQR)).

Results varied widely in studies with active surveillance that reported specific AEs after treatment of Mf positive persons. Rates of fever were 100% in some studies, and other systemic symptoms including myalgia/arthralgia, dizziness, giddiness, weakness, fatigue, and malaise were also very common. Rates of systemic events in amicrofilaremic individuals were much lower (**Fig 4**). The rates and patterns of AEs did not clearly differ between DEC and IVM, but AEs were much less common after ALB, and they were low with any medication in amicrofilaremic participants (**Fig 5**).

## Discussion

In this review we initially sought to quantify the effects of various factors on AE rates that occur following MDA for LF. We quickly realized that the heterogeneity in the way AEs have been reported in the literature would not allow a meaningful quantitative multivariate analysis. We nevertheless felt a compilation of reported AE rates would be beneficial. Despite the limitations of combining data from methodologically disparate studies, we believe the compiled data illustrate the following main points: 1) AEs are very common in microfilaremic patients after single-dose treatment of LF with drugs (IVM and DEC) that rapidly reduce Mf counts. 2) AEs are much less common in amicrofilaremic participants, regardless of treatment regimen. 3) Passive surveillance tends to underestimate the occurrence of AEs, and 4) Heterogeneity in the stringency of AE surveillance and format of AE reporting makes comparisons between studies difficult.

The relationship between AE rates and the prevalence of microfilaremia is illustrated by the striking differences between study arms with 100% microfilaremia and those with no microfilaremia. It would have been interesting to compare Mf prevalence to AE rates among the community studies with varied Mf prevalence; this was not attempted because of the variability in AE reporting for these studies and because uncertainty regarding true Mf rates would have made this comparison unreliable.

It is clear that community proclivities for reporting AEs vary from place to place and study to study. This is perhaps most evident in reported AE rates after placebo treatment. Studies with highly active AE surveillance in Haiti and Tahiti reported high AE rates after placebo treatment [54, 84, 93], but AE rates were low after placebo treatment in Ghana and India [86, 120]. This place-to-place AE reporting variability is also evident in the wide range of AE rates reported among different studies with the same treatment regimens (see Figs 3 and 5). Potential reasons for this might include the prevalence of STH or other helminth infections, differing intensities of LF infection, and varying cultural norms. In addition, where populations have been sensitized to expect AEs following MDA, more AEs may be perceived [18].

Nearly all the studies cited in this review reported AE rates in some manner, but we were only able to include 55 in the quantitative synthesis. The primary reason for excluding studies was that they did not present AE data in a way that linked AE rates to treatment regimens. For example, many studies reported AE severity scores rather than rates. Others reported that AE rates did not differ significantly between treatment groups, but did not report the numbers for each group. When AE rates were reported by treatment group, comparisons were often hampered by non-standardized AE reporting procedures. Some authors did not report the timeframe over which AE surveillance was conducted, making it difficult to surmise whether early or late AEs may have been missed. Although most studies included in our analysis described whether AE surveillance was active or passive, many contained insufficient detail to determine how sensitive the study procedures were for detecting AEs. For example, ascertainment rates (the proportion of participants in community-based studies who were actually visited and queried about AEs) were almost never reported.

This review has several strengths and weaknesses. The primary strength is that it compiles data from 30 years of published studies. It also illustrates how variable AE reporting can be, and it provides a context for interpreting AE rates observed in future LF treatment studies. One weakness was our inability to include data from many high quality studies that did not report AEs by treatment arm. In addition, because we restricted our analysis to studies of single-dose therapy, many rich and highly informative studies that used multi-dose treatment regimens were excluded. In general, the pattern of AEs reported in such studies was similar to single dose studies. That is to say, the rate and severity of AEs increased with increasing Mf counts and most AEs occurred during the first 48 hours after the initial treatment dose [38, 82].

The heterogeneity in AE reporting among the reviewed studies highlights the need for a more structured approach to AE reporting in LF treatment studies. Although this problem is not unique to filariasis [128], it can be compounded by the nature of community-based studies. We therefore suggest the following measures for improving AE reporting in community based treatment trials for LF and other neglected tropical diseases (Box 1). **1) Clearly specify the methods for ascertaining AEs.** Indicate whether an attempt was made to contact each participant (active surveillance) or whether participants were required to seek out study staff to report AEs (passive surveillance). Indicate when and how often participants were contacted. Avoid ambiguous language such as “followed closely”, or “closely monitored”. Rather, describe what was actually done. For example, “treated individuals were visited daily in their homes for five days after treatment”. **2) If surveillance was active, report the ascertainment rate;** that is, the proportion of participants sought during surveillance that was actually found. Knowing what proportion of participants actually contributed to the reported AE rates will help the reader assess the reported findings. For example, one study reported, “All subjects [were] asked to come to the study site on day 2 and day 5….In addition, team members also went door to door.” The door to door contacting was presumably meant to ascertain AEs in subjects not reporting at the study site, since subjects may choose not to present for follow-up either because they feel well and see no need, or because they feel ill and don’t wish to leave their homes. The higher the proportion of participants for whom actual AE status is not ascertained, the greater the uncertainty regarding the reported AE rates. Unfortunately, the study cited—and most other community studies we reviewed—did not report ascertainment rates. **3) Report numerators and denominators.** When severity scores are used (for example, 1 = mild, 2 = moderate, 3 = severe) to compare AEs between study groups, the actual number of persons experiencing AEs should also be reported so that rates can be calculated. The difference between one person with a severe AE and three people with one mild AE each is important, and the reporting of AE data should allow the reader to distinguish the difference. In addition to clearly specifying the number of persons experiencing AEs (the numerator), the denominator should be clearly defined. In active studies, we suggest reporting AE rates as the proportion of those experiencing AEs over the number actually assessed. For example, five persons experiencing AEs among 20 patients treated should be reported as 50% (not 25%) if only 10 of those treated were actually assessed. **4) Use standardized grading criteria** for reporting AE severity. Examples include the National Cancer Institute’s Common Terminology Criteria for Adverse Events (available at https://evs.nci.nih.gov/ftp1/CTCAE/About.html) or the Division of AIDS Table for Grading the Severity of Adult and Pediatric Adverse Events (available at http://rsc.tech-res.com/clinical-research-sites/safety-reporting/daids-grading-tables). **5) Follow CONSORT guidelines** for better reporting of harms in clinical trials [129].

**Box 1. Recommendation for AE reporting in filariasis studies.**

- Clearly specify the method for ascertaining AEs.
  ○ Was an attempt made to contact each participant to assess for AEs?
  ○ When and how often were AEs assessed?
- Report the ascertainment rate (the percentage of participants assessed for AEs).
- Report numerators and denominators.
  ○ How many adverse events occurred among how many participants?
  ○ Report the actual numbers, even if severity scores are used, to allow readers to calculate rates.
- Use standardized grading for reporting AE severity.
- Follow CONSORT guidelines for reporting harms.

In conclusion, this review has shown that AEs following single dose treatment of LF are common and should be expected in microfilaremic patients. This information provides a useful context for understanding AEs observed with new treatments for LF. Clear and detailed reporting of AEs in community treatment studies is essential to accurately inform elimination program workers and their communities, and to set appropriate expectations. The fear of MDA-associated AEs is often out of proportion to the actual risk, because most post-treatment AEs are mild and transient. A frank explanation of AEs as a marker for treatment efficacy by program managers and community health workers may improve compliance with MDA and facilitate LF elimination efforts.

## Supporting information

S1 Table PRISMA checklist. (DOC)

S2 Table Studies groups and data included in quantitative analysis. (XLSX)

S1 Fig PRISMA flow diagram. Please note that Fig 1 contains the same information in a slightly modified format. (DOC)
