# Supplementary material for: Adverse events following single dose treatment of lymphatic filariasis: Observations from a review of the literature
Source: PLoS Negl Trop Dis. 2018 May 16;12(5):e0006454. doi: 10.1371/journal.pntd.0006454 (PMC5973625; doi:10.1371/journal.pntd.0006454)
Supplement: S1 Fig — (DOC) [file pntd.0006454.s003.doc]

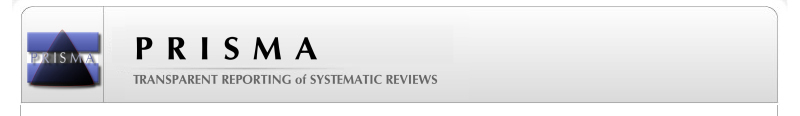
**PRISMA 2009 Flow Diagram**

**Screening**

**Included**

**Eligibility**

**Identification**

Records identified through database searching
(n = 147)

Additional records identified through other sources
(n = 96)

Records after duplicates removed
(n = 231 )

Records screened
(n = 231 )

Records excluded
(n = 72 )

Full-text articles assessed for eligibility
(n = 162 )

Full-text articles excluded (n = 107 )

46 Insufficient AE reporting

14 No primary data

14 Not single-dose treatment

14 AE not reported by treatment group

10 Retrospective surveys

7 Dates outside inclusion range

2 No IDA medications

Studies included in qualitative synthesis
(n = 55 )

Studies included in quantitative synthesis (meta-analysis)
(n = 55 )
